# PSMA expression: a potential ally for the pathologist in prostate cancer diagnosis

**DOI:** 10.1038/s41598-018-22594-1

**Published:** 2018-03-09

**Authors:** Sara Bravaccini, Maurizio Puccetti, Martine Bocchini, Sara Ravaioli, Monica Celli, Emanuela Scarpi, Ugo De Giorgi, Maria Maddalena Tumedei, Giandomenico Raulli, Loredana Cardinale, Giovanni Paganelli

**Affiliations:** 1 Biosciences Laboratory, Istituto Scientifico Romagnolo per lo Studio e la Cura dei Tumori (IRST) IRCCS, Meldola, Italy; 2 Pathology Unit, Santa Maria delle Croci Hospital, Ravenna, Italy; 3 Nuclear Medicine Unit, Istituto Scientifico Romagnolo per lo Studio e la Cura dei Tumori (IRST) IRCCS, Meldola, Italy; 4 Unit of Biostatistics and Clinical Trials, Istituto Scientifico Romagnolo per lo Studio e la Cura dei Tumori (IRST) IRCCS, Meldola, Italy; 5 Department of Medical Oncology, Istituto Scientifico Romagnolo per lo Studio e la Cura dei Tumori (IRST) IRCCS, Meldola, Italy

## Abstract

Prostate cancer (PCa) patients are risk-stratified on the basis of clinical stage and PSA level at diagnosis and the Gleason Score (GS) in prostate biopsy. However, these parameters are not completely accurate in discriminating between high- and low-risk disease, creating a need for a reliable marker to determine aggressiveness. Prostate-specific membrane antigen (PSMA) appears to fulfill this need. We analyzed 79 prostate biopsies and 28 prostatectomies to assess whether PSMA expression detected by immunohistochemistry is related to GS. PSMA expression was correlated with GS in both sample types (biopsies, P < 0.0001 and prostatectomy samples, P = 0.007). We observed lower PSMA expression in Gleason pattern 3 than Gleason pattern 4, suggesting that this biomarker could be useful to distinguish between these entities (p < 0.0001). The best cut-off value of 45% immunopositivity was determined by receiver operating characteristic (ROC) curve analysis. In Gleason pattern 3 *vs*. Gleason pattern 4 and 5, PSMA sensitivity was 84.1% (95% CI 76.5%-91.7%) and specificity was 95.2% (95% CI 90.6%-99.8%), with an area under the curve of 93.1 (95% CI 88.8–97.4). Our results suggest that PSMA represents a potential ally for the pathologist in the diagnostic work-up of PCa to overcome long-standing morphological classification limits.

## Introduction

Prostate cancer (PCa) is the second most common cancer among males. In 2012 an estimated 1.1 million men worldwide were diagnosed with the disease, 70% (759,000) in (more) developed countries^1^. Many patients are diagnosed with clinically non-significant or indolent PCa. As far as organ-confined PCa is concerned, tumor grade remains the main determinant of PCa biological behavior and is thus widely used in risk-stratification algorithms to guide clinicians in their choice of treatment (*i.e*. active surveillance or curative surgery and/or radiation therapy). PCa grading is usually established by the Gleason Score (GS), a system that sums the differentiation scores of the 2 most represented histological tumor architectures observed in core biopsy/surgical specimens. However, GS evaluation has some limitations, namely suboptimal inter-observer reproducibility and an arduous subclassification of GS 7 lesions (*i.e*. GS 3 + 4 *vs*. GS 4 + 3 lesions) due mainly to the difficulty in quantifying the most represented histological component (Gleason pattern 3 *vs*. Gleason pattern 4). For these reasons, tumor differentiation remains challenging.

In organ-confined PCa, both GS and serum prostate-specific antigen (PSA) level contribute to defining patient treatment and follow-up. Nonetheless, serum PSA is burdened by low accuracy because it is an organ-specific rather than tumor-specific biomarker and can also show an abnormal increase in the presence of benign prostatic hyperplasia. This is one of the most important limitations of PSA for diagnostic/prognostic purposes^2,3^.

The International Society of Urological Pathology (ISUP) has issued guidelines for PCa grading and outcome prediction^4^. In particular, it is important to discriminate between patients with overall low-risk PCa (GS < 6/ISUP group 1, with PSA < 10 ng/mL and/or T1-T2a) who may benefit from treatment deferral (*i.e*. active surveillance) and those with intermediate-risk PCa (GS 7, defined as either 3 + 4, ISUP group 2, or 4 + 3, ISUP group 3, and/or T2b) who require active treatment^4^. Patients with the highest GS score (8–10) are classified as Grade Group 4 (GS 8) or Grade Group 5 (GS 9 and 10)^4^. Thus, there is clearly an urgent need for more accurate and reliable prognostic markers capable of distinguishing patients who require intensive treatment from who are candidates for a watch-and-wait approach.

Prostate-specific membrane antigen (PSMA) is a non-soluble type 2 integral membrane protein with carboxypeptidase activity, expressed on the apical surface of endothelial cells^5–10^. It is weakly expressed in normal prostate tissue but strongly upregulated in prostate cancer^10^. In actual fact, tissue expression of this antigen is not fully prostate-specific as it is also expressed in the neovasculature of numerous solid malignancies^11^. PSMA overexpression is associated with higher PCa grade and androgen deprivation, further increasing in metastatic disease and when castration resistance sets in. This suggests that PSMA plays a functional role in PCa progression, but the correlation with GS and serum PSA is not well established as yet^12,13^.

Given its biological features, PSMA is also currently being validated as a PET imaging biomarker for primary PCa localization, lesion grading and primary staging. The most widely used PSMA-ligand for human PET imaging is the low-weight urea-based PSMA inhibitor Glu-NH-CO-NH-Lys(Ahx)-HBED-CC labelled with ^68^Gallium (*i.e*. ^68^Ga-PSMA-11). Like the PSMA monoclonal antibody used for immunostaining in our study (*i.e*. SP29), ^68^Ga-PSMA-11 binds to a C-terminal epitope of the large extra-membrane domain of PSMA. Early clinical experiences in primary PCa staging have shown that ^68^Ga-PSMA-11 PET has 67% sensitivity, 92% specificity, a 97% positive predictive value, a 42% negative predictive value, and 72% accuracy. The degree of uptake of ^68^Ga-PSMA-11 in PCa cells (*i.e*. PCa lesion SUVmax) has been found to significantly correlate with PSMA expression in lesions, measured by immunohistochemistry and GS. Lymph node staging using ^68^Ga-PSMA-11 PET has shown a sensitivity, specificity, and accuracy of 77.9%, 97.3% and 89.9%, respectively^14–20^. The same studies also reported that about 8% of PCa do not overexpress PSMA^14–20^.

The aim of the present study was to assess whether PCa patients can be stratified on the basis of PSMA expression and, in particular, to establish whether its expression is related to GS and serum PSA values at diagnosis.

## Results

**Figure 1:**
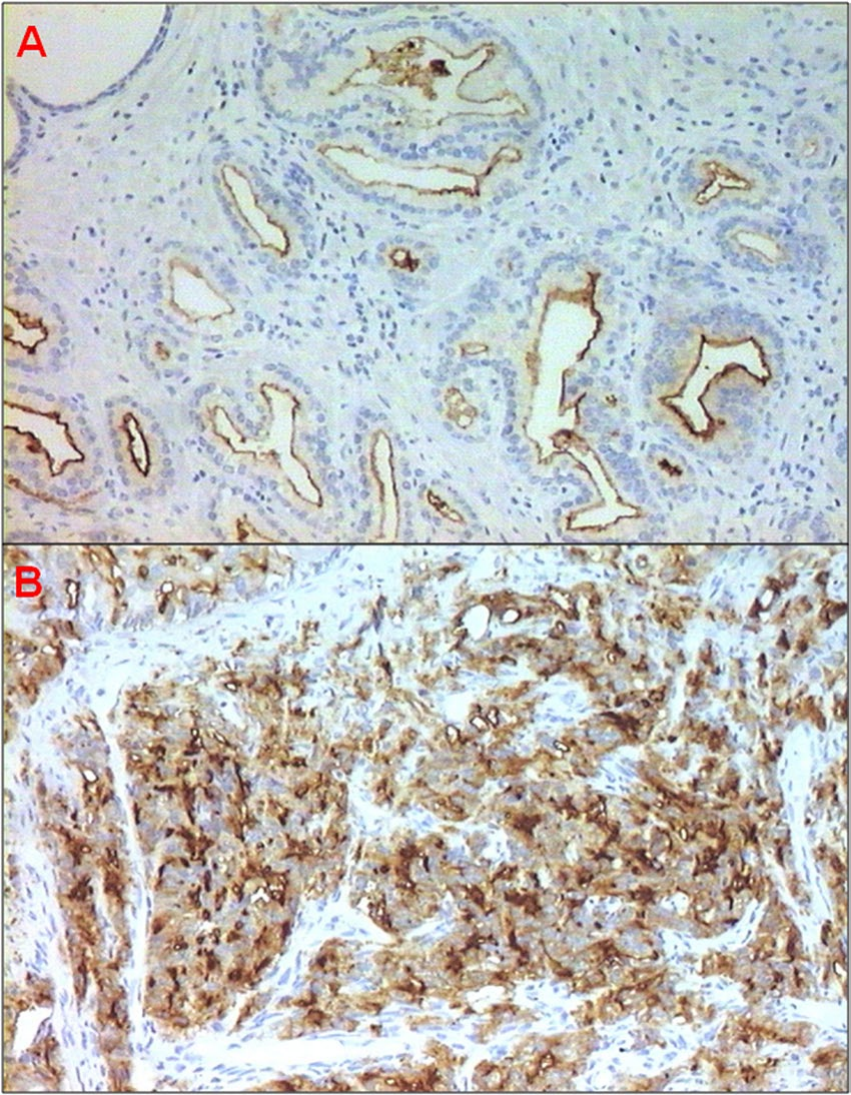
PSMA-positive PCa. (**A**) GS 6 (3 + 3, Grade Group 1) prostate adenocarcinoma (10 × magnification) with moderate endoluminal membrane staining. (**B**) GS 8 (4 + 4, Grade Group 4) prostate adenocarcinoma (10 × magnification) with strong membrane staining.

The analysis of PSMA expression was feasible in the overall series of biopsies and prostatectomies. Nineteen bioptic specimens were classified as Grade Group 1 (GS 6), 35 as Grade Group 2–3 (GS 7), 25 as Grade Group 4–5 (GS 8–10). With regard to the 28 prostatectomies, 6 lesions were Grade Group 1 (GS 6), 19 were Grade Group 2–3 (GS 7) and 3 were Grade Group 4–5. The median PSMA expression in terms of percentage and H-score differed significantly and positively correlated with GS in both biopsies (P < 0.0001) and prostatectomies (P = 0.007) (Fig. 1) (Table 1). The same correlation in terms of PSMA staining intensity was observed in both biopsies and prostatectomy specimens (P < 0.0001 and P = 0.006, respectively) (Table 2). The median value of PSMA expression was similar in the small series of Grade Group 2 and Grade Group 3 lesions analyzed (Table 3).

**Table 1:** Median PSMA expression values in the different Grade Groups.

Median values of PSMA expression %* (range)				
Grade Group	No.	Biopsy	No.	Prostatectomy
**1**	19	20 (0–60)	6	15 (0–40)
**2-3**	35	43 (0–80)	19	40 (1–65)
**4-5**	25	70 (0–100)	3	80 (50–90)
		**P** < **0.0001**		**P** = **0.007**
**Median values of PSMA expression H-score** ^#^ **(range)**				
**1**	19	20 (0–120)	6	35 (0–80)
**2-3**	35	90 (0–210)	19	80 (1–195)
**4-5**	25	210 (0–300)	3	240 (150–270)
		**P** **<** **0.0001**		**P** = **0.007**

^*^Percentage of immunopositive tumor cells.

^#^H-score, defined as the product of the percentage of the immunopositive tumor cells and the staining intensity.

**Table 2:** PSMA staining intensity in the different Grade Groups.

Grade Group					
PSMA staining intensity^*^	1	2-3	4-5	Total	P
	No. (%)	No. (%)	No. (%)	No.	
***Biopsy***					
**0**	6 (66.7)	2 (22.2)	1 (11.1)	9	
**1**	5 (71.4)	2 (28.6)	0	7	
**2**	7 (20.6)	22 (64.7)	5 (14.7)	34	
**3**	1 (3.5)	9 (31.0)	19 (65.5)	29	**<0.0001**
***Prostatectomy***					
**0**	2 (100)	0	0	2	
**1**	1 (20.0)	4 (80.0)	0	5	
**2**	2 (13.3)	13 (86.7)	0	15	
**3**	1 (16.7)	2 (33.3)	3 (50.0)	6	**0.006**

^*^0 absent, 1 weak, 2 moderate, 3 strong PSMA staining intensity.

**Table 3:** Median values of PSMA expression in Grade Group 2 and 3 lesions.

	No.	PSMA expression (%)^*^		PSMA expression (H score)^#^	
		Median value (range)	P	Median value (range)	P
***Overall series***					
Grade Group 2	33	40 (15–80)		80 (15–195)	
Grade Group 3	21	40 (0–70)	0.562	80 (0–210)	0.600
***Biopsy***					
Grade Group 2	19	45 (20–80)		90 (40–160)	
Grade Group 3	16	41.5 (0–70)	0.469	88 (0–210)	0.610
***Prostatectomy***					
Grade Group 2	14	37.5 (15–65)		75 (15–195)	
Grade Group 3	5	40 (1–53)	0.890	80 (1–106)	0.679

^*^Percentage of immunopositive tumor cells.

^#^H-score, defined as the product of the percentage of the immunopositive tumor cells and the staining intensity.

Table 4 reports median PSMA expression values in the different Gleason patterns according to cellular morphology and differentiation. Lower expression of PSMA was observed in Gleason pattern 3 with respect to Gleason patterns 4 and 5, both of which showed higher PSMA expression (P < 0.0001) (Table 4). Absent or weak PSMA expression was observed in the normal and benign tissue analyzed (Table 4). In addition, stronger PSMA staining intensity was more frequently observed in the less differentiated Gleason patterns 4 and 5 than in Gleason pattern 3 (p < 0.0001) (Table 5).

**Table 4:** PSMA expression in the different Gleason patterns and non malignant tissue.

	No.^†^	PSMA expression (%)*		PSMA expression (H-score)^#^	
		Median value (range)	P	Median value (range)	P
Non malignant tissue^§^	43	0 (0–60)		0 (0–150)	
Pattern 3	83	10 (0–70)		10 (0–120)	
Pattern 4	75	70 (0–100)		210 (0–300)	
Pattern 5	13	90 (40–100)	**<0.0001**	270 (120–300)	**<0.0001**

^*^Percentage of immunopositive tumor cells.

^#^H-score, defined as the product of the percentage of the immunopositive tumor cells and the staining intensity.

^†^Number of foci analyzed.

^§^Normal tissue, benign prostatic hyperplasia.

**Table 5:** PSMA expression staining intensity in the different Gleason patterns non malignant tissue.

PSMA staining intensity^#^	Non malignant tissue*	Pattern 3	Pattern 4	Pattern 5	P
	No. (%)	No. (%)	No. (%)	No. (%)	
0	28 (82.4)	31 (37.4)	5 (6.7)	0	
1	1 (2.9)	28 (33.7)	2 (2.7)	0	
2	3 (8.8)	20 (24.1)	8 (10.6)	0	
3	2 (5.9)	4 (4.8)	60 (80.0)	13 (100)	**<0.0001**

^*^Normal tissue, benign prostatic hyperplasia.

^#^0 absent, 1 weak, 2 moderate, 3 strong PSMA staining intensity.

The sensitivity and specificity of PSMA expression according to the best cut-off value of 45% determined by the Receiver Operating Characteristic (ROC) curve analysis were 84.1% (95% confidence intervals [CI] 76.5%-91.7%) and 95.2% (95% CI 90.6%-99.8%) respectively, in distinguishing Gleason pattern 3 from Gleason patterns 4 and 5, with an area under the curve (AUC) of 93.1 (95% CI 88.8–97.4).

The overall accuracy of PSMA expression to classify Grade Group 1 and 2 lesions *vs*. Grade Groups 3, 4 and 5 lesions (at the best cut-off value of 45%) was 72.9% (95% CI 64.5–81.3), with a sensitivity of 82% (95% CI 68–96) and a specificity of 70% (95% CI 59–80).

Similar PSMA expression (%, H-score and staining intensity) was observed in biopsies and prostatectomies of the 23 patients for whom data on prostatectomy were also available (Tables 6 and 7).

**Table 6:** Median PSMA expression values in biopsy and prostatectomy in terms of percentage and H-score.

	PSMA expression (%)*		PSMA expression (H score)^#^	
	Median value (range)	P	Median value (range)	P
Biopsy	45 (0–80)	0.447	90 (0–210)	
Prostatectomy	40 (0–90)		80 (0–270)	0.453

^*^Percentage of immunopositive tumor cells.

^#^H-score, defined as the product of the percentage of the immunopositive tumor cells and the staining intensity.

**Table 7:** PSMA expression staining intensity in biopsy and prostatectomy.

PSMA staining intensity^*^	Biopsy	Prostatectomy	
	No. (%)	No. (%)	P
0	4 (17.4)	2 (8.7)	
1	2 (8.7)	4 (17.4)	
2	13 (56.5)	12 (52.2)	
3	4 (17.4)	5 (21.7)	0.627

^*^0 absent, 1 weak, 2 moderate, 3 strong PSMA staining intensity.

**Figure 2:**
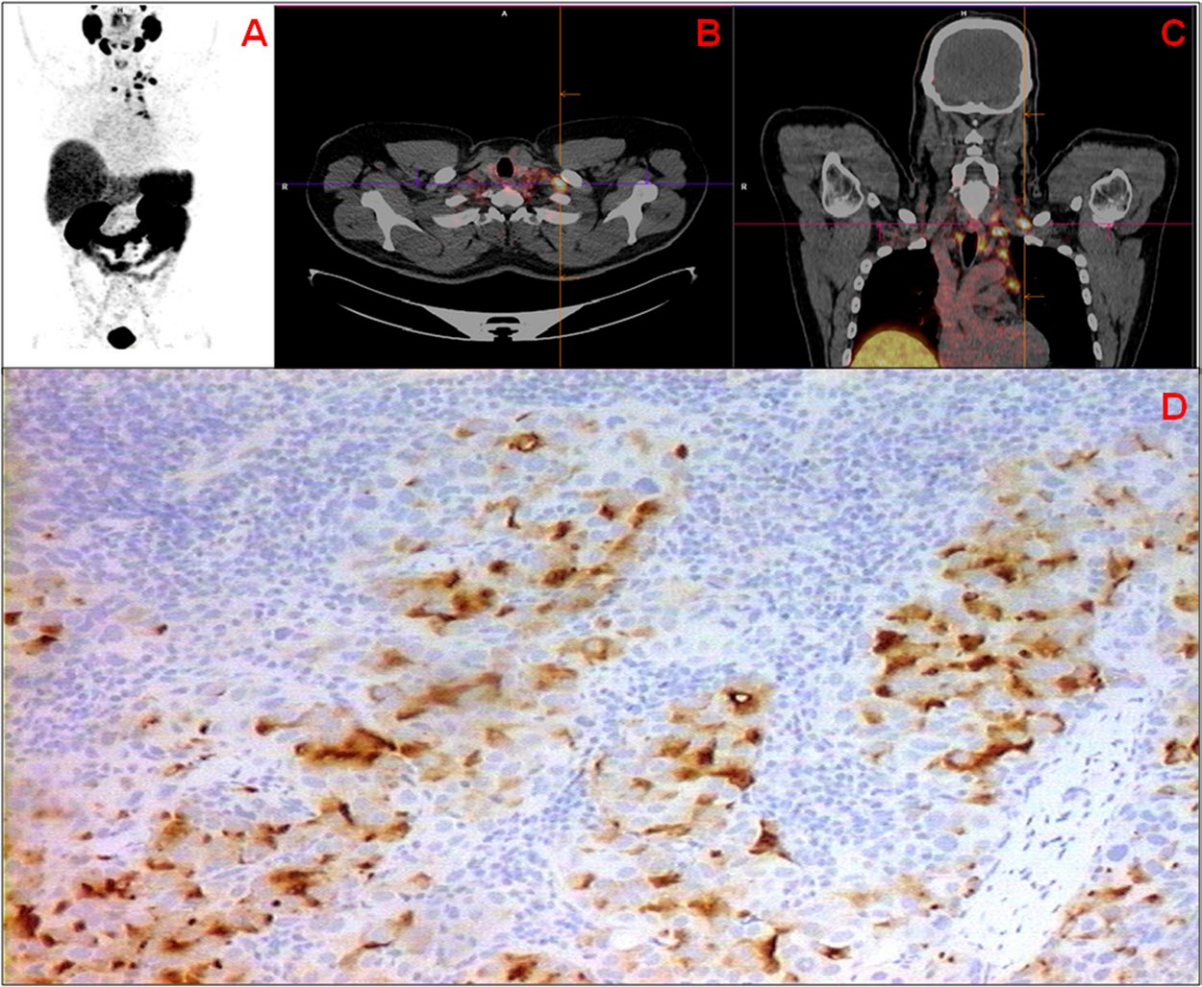
Restaging of a 46-year-old male with biochemical relapse after radical prostatectomy for high-risk PCa (Grade Group 4; PSA at diagnosis: 67.0 ng/mL). (**A**) PSMA PET MIP (maximum intensity projection) visualization showing PSMA-avid lymphadenopathy above and below the diaphragm. (**B**,**C)** PSMA PET/CT (transaxial and coronal views) showing intense PSMA uptake in a left retroclavicular node (SUVmax: 12.8). (**D**) PSMA expression in PCa cells in lymph node biopsy of the same patient (20 × magnification).

PSMA expression was positively correlated with basal PSA serum value with respect to both percentage and H-score (Table 8). The median PSA value was 7.85 ng/mL (range 3.2–452.4 ng/mL). No correlation was found between PSMA expression and age. Figure 2 shows ^68^Ga-PSMA PET/CT images of *in vivo* PSMA expression in a 46-year-old male with biochemical relapse after radical prostatectomy for high-risk PCa (Gleason 4 + 4). Figure 2D shows PSMA expression in PCa cells in lymph node biopsy of the same patient.

**Table 8:** Correlation between PSMA expression and baseline PSA value and age.

	PSA		Age	
	r_s_	P	r_s_	P
PSMA expression (%)*	0.35	0.003	−0.03	0.810
PSMA expression (H-score)^#^	0.36	0.002	−0.04	0.708
Baseline PSA	—	—	0.22	0.056

^*^Percentage of immunopositive tumor cells.

^#^H-score, defined as the product of the percentage of the immunopositive tumor cells and the staining intensity.

## Discussion

The clinical management of patients with PCa is normally based on serum PSA value, Gleason score and clinical stage^2^. However, these parameters are not 100% accurate in discriminating between low- and high-risk patients, indicating the need for companion biomarkers to better predict disease evolution and improve patient stratification. Although PSMA is a promising biomarker in that it is more specific than PSA, its role in PCa diagnosis and evolution has yet to be confirmed^2,3^. In fact, differences in PSMA expression in terms of cellular or tissue localization may depend on the type of antibody used or sample analyzed. It has also been shown that healthy and benign tissues display PSMA positivity in the cytoplasm and vasculature^11,21,22^. We observed a lower PSMA expression in the well-differentiated Gleason patterns 3 than 4, indicating that this biomarker could be useful to distinguish between these 2 challenging morphological entities.

Despite the retrospective nature of the study and the limited number of cases analyzed, we found that PSMA expression in terms of percentage, H-score and intensity may be related to GS, reflecting tumor aggressiveness in both bioptic and radical prostatectomy specimens. This explorative work could thus form the basis for future research to assess the real diagnostic and prognostic value of PSMA. It would also be interesting to see whether PSMA is capable of distinguishing between high-grade prostate intraepithelial neoplasia (PIN), intraductal carcinoma and atypical small acinar proliferation (ASAP).

It is known that GS 3 + 4 lesions are less aggressive than GS 4 + 3 lesions, and several studies have assessed the differences between these 2 subtypes by integrating GS with other prognostic factors and biochemical progression^23,24^. In 2014, a new grading system consisting of 5 prognostically distinct Grade Groups was proposed on the basis of data from Johns Hopkins Hospital. This new classification system partitions the old Gleason score 7 into 2 distinct groups, *i.e*. Grade Group 2 (Gleason score 3 + 4) and Grade Group 3 (Gleason score 4 + 3), with studies confirming a significantly poorer prognosis for the latter group^4^. This finding takes on an even greater importance when a GS 3 + 4 lesion is underestimated because the evaluation of the GS depends entirely on the pathologist’s expertise^24^. In addition, it is not always easy to quantify and distinguish the proportion of Gleason patterns 3 and 4 in prostate biopsy specimens.

Although we did not observe a significant difference between Grade Group 2 and 3 lesions in terms of PSMA expression, our results highlighted the promising role of PSMA in discriminating between well differentiated Gleason patterns 3 and 4 within the same sample.

Although a biopsy generally does not reflect the histology of the entire lesion, it may result in the complete removal of a tumor. Our results showed good concordance in PSMA expression between biopsy and prostatectomy. In agreement with some authors who studied PSMA expression in benign lesions and PCa, we demonstrated that PSMA expression reflects the aggressiveness of the disease^13,25,26^. We also focused our analysis on PSMA expression between Gleason patterns 3 and 4.

The positive correlation between PSMA expression and baseline PSA serum levels makes it a reliable biomarker for clinical decision making.

It can also be hypothesized that different tumor uptake of ^68^Ga-PSMA may reflect PSMA expression status. This information could be used to distinguish more aggressive lesions from indolent ones in diagnosis and therapy, as tumors with high PSMA expression may have a high uptake of radiolabeled PSMA ligands. Other markers of aggressive variants of PCa such as those associated with neuroendocrine differentiation (PTEN, p53, BRCA2) could be compared with PSMA expression in patients with advanced disease with poor prognostic features or in those with visceral metastases to have a better overall picture of the biological and clinical impact of PSMA expression^27–29^. Furthermore, the use of ^68^Ga-PSMA PET/CT as a potential biomarker for the *in vivo* assessment of PSMA expression could provide reliable information on prognosis and prediction of response to different antitumor agents in advanced PCa, as recently seen for 18F-choline PET/CT^30–32^, opening up new avenues of research into PSMA pathological-clinical correlations.

PSMA could prove to be a powerful ally for pathologists in the diagnostic workup of PCa. It could also facilitate the selection of candidates for surveillance/observation programs or local treatment (*e.g*. prostatectomy or radiotherapy), thus reducing healthcare costs and the risk of overtreatment. Our hypothesis, if confirmed in larger prospective trials, could make a positive impact on/lead to an important change the clinical workup of PCa patients.

## Methods

### Case series

This exploratory retrospective study included 84 patients with acinar adenocarcinoma of the prostate followed at the Department of Urology of Santa Maria delle Croci Hospital in Ravenna (Italy) from 2013 to 2017. Twenty-three (29%) underwent both prostate biopsy and prostatectomy. Overall, 79 biopsies and 28 prostatectomies were analyzed. The study protocol was reviewed and approved by IRST and AVR (Area Vasta Romagna) Ethics Committee (approval no 1478, 11-Nov-2015). Median age was 64 years (range 46 to 84 years). All of the analyses were carried out in accordance with the relevant guidelines and regulations, and written informed consent was obtained from all study participants. Formalin-fixed paraffin-embedded samples were used for diagnosis. The histology and grading of prostate lesions were established by expert pathologists at the Ravenna hospital in accordance with International Society of Urological Pathology (ISUP) Consensus Conference guidelines^4^.

Given that, at the time of the study ^68^Ga-PSMA11 PET/CT was only performed at our institute in patients with biochemical recurrence, we provide the images of a patient that underwent ^68^Ga-PSMA 11 PET/CT for biochemical recurrence in relation to PSMA expression detected in the lymph node biopsy.

### Immunohistochemistry

Immunostaining for PSMA expression was performed using the Ventana Benchmark XT staining system (Ventana Medical Systems, Tucson, AZ, USA) with Optiview DAB Detection Kit (Ventana Medical Systems). Tissue sections were incubated for 32 minutes with ready-to-use anti-PSMA antibody (SP29 Spring Bioscience, Pleasanton, CA, USA). Sections were automatically counterstained with hematoxylin II (Ventana Medical Systems). PCa and breast cancer tissues were used as positive and negative controls, respectively, in all of the experiments. Biomarker expression was quantified as the percentage of tumor cells with membrane staining out of the total number of tumor cells. Non malignant tissue around the tumor, when present, was also evaluated for PSMA expression. Staining intensity (*i.e*. 0 absent, 1 weak, 2 moderate, 3 strong) was assessed to calculate the H-score, defined as the product of the percentage of the immunopositive tumor cells and the staining intensity^33,34^. Given that there is still no established cut-off value for PSMA expression, we chose a cut-off of 45% immunopositive tumor cells on the basis of the results from ROC curve analysis. All samples were evaluated by 2 independent observers. Disagreement of >10% positive cells was resolved by consensus after joint review using a multihead microscope.

### Statistical analysis

Descriptive statistics are reported as counts, proportions, median values and ranges. The Chi-square test was used to determine the strength of the association between categorical variables. The relationship between median PSMA expression values and GS Nonparametric was evaluated by ranking statistics (Wilcoxon median test), and the Kruskall-Wallis test was used to assess the relationship between PSMA expression values in the different histological patterns. Spearman’s rank correlation test was used to investigate the relation between PSMA expression and PSA serum level and age. All P values were based on two-sided testing and values < 0.05 were considered statistically significant. All the statistical analyses were carried out using SAS Software, version 9.4 (SAS Institute, Cary, NC, USA).

### Data sharing statement

All data generated or analyzed during this study are included in this manuscript.

## References

[1] http://globocan.iarc.fr/old/FactSheets/cancers/prostate-new.asp, GLOBOCAN2012 (IARC), Section of Cancer Surveillance (20/2/2018).

[2] Ross T, Ahmed K, Raison N, Challacombe B, Dasgupta P (2016). Clarifying the PSA grey zone: The management of patients with a borderline PSA. Int J Clin Pract..

[3] Ezenwa EV (2012). The value of percentage free prostate specific antigen (PSA) in the detection of prostate ancer among patients with intermediate levels of total PSA (4.0-10.0 ng/mL) in Nigeria. Arab J Urol..

[4] Epstein JI (2016). The 2014 International Society of Urological Pathology (ISUP) Consensus Conference on Gleason Grading of Prostatic Carcinoma: Definition of Grading Patterns and Proposal for a New Grading System. Am J Surg Pathol..

[5] Israeli RS, Powell CT, Corr JG, Fair WR, Heston WD (1994). Expression of the prostate-specific membrane antigen. Cancer Res..

[6] Wright GL, Haley C, Beckett ML, Schellhammer PF (1995). Expression of prostate-specific membrane antigen in normal, benign, and malignant prostate tissues. Urol Oncol..

[7] Troyer JK, Beckett ML, Wright GL (1995). Detection and characterization of the prostate-specific membrane antigen (PSMA) in tissue extracts and body fluids. Int J Cancer..

[8] Sokoloff R, Norton KC, Gasior CL, Marker KM, Grauer LS (2000). A dual-monoclonal sandwich assay for prostate-specific membrane antigen: levels in tissues, seminal fluid and urine. Prostate..

[9] Bostwick DG, Pacelli A, Blute M, Roche P, Murphy GP (1998). Prostate specific membrane antigen expression in prostatic intraepithelial neoplasia and adenocarcinoma: a study of 184 cases. Cancer..

[10] Silver DA, Pellicer I, Fair WR, Heston WD, Cordon-Cardo C (1997). Prostate-specific membrane antigen expression in normal and malignant human tissues. Clin Cancer Res..

[11] Chang SS (1999). Five different anti-prostate-specific membrane antigen (PSMA) antibodies confirm PSMA expression in tumor-associated neovasculature. Cancer Res..

[12] Wright GL (1996). Upregulation of prostate-specific membrane antigen after androgen- deprivation therapy. Urology..

[13] Sweat SD, Pacelli A, Murphy GP, Bostwick DG (1998). Prostate-specific membrane antigen expression is greatest in prostate adenocarcinoma and lymph node metastases. Urology..

[14] Eiber M (2016). Simultaneous (68)Ga-PSMA HBED-CC PET/MRI Improves the Localization of Primary Prostate Cancer. Eur Urol..

[15] Maurer T (2016). Diagnostic Efficacy of 68Gallium-PSMA-PET compared to conventional imaging in lymph node staging of 130 consecutive patients with intermediate to high-risk prostate cancer. J Urol..

[16] Budäus L (2015). Initial experience of (68)Ga-PSMA PET/CT imaging in high-risk prostate cancer patients prior to radical prostatectomy. Eur Urol..

[17] Fendler WP (2016). 68Ga-PSMA-HBED-CC PET/CT detects location and extent of primary prostate cancer. J Nucl Med..

[18] Woythal N (2018). Immunohistochemical validation of PSMA-expression measured by (68)Ga-PSMA PET/CT in primary prostate cancer. J Nucl Med..

[19] Uprimny C (2017). 68Ga-PSMA-11 PET/CT in primary staging of prostate cancer: PSA and Gleason score predict the intensity of tracer accumulation in the primary tumour. Eur J Nucl Med Mol Imaging.

[20] Weineisen M (2015). 68Ga- and 177Lu-labeled PSMA I&T: optimization of a PSMA-targeted theranostic concept and first proof-of-concept human studies. J Nucl Med..

[21] Chang SS (2004). Overview of prostate-specific membrane antigen. Rev Urol..

[22] Mhawech-Fauceglia P (2007). Prostate-specific membrane antigen (PSMA) protein expression in normal and neoplastic tissues and its sensitivity and specificity in prostate adenocarcinoma: an immunohistochemical study using multiple tumour tissue microarray technique. Histopathology..

[23] Coley RY, Zeger SL, Mamawala M, Pienta KJ, Carter HB (2017). Prediction of the pathologic gleason score to inform a personalized management program for prostate cancer. Eur Urol..

[24] Coard KC, Freeman VL (2004). Gleason grading of prostate cancer: level of concordance between pathologists at the University Hospital of the West Indies. Am J Clin Pathol..

[25] Marchal C (2004). Expression of prostate specific membrane antigen (PSMA) in prostatic adenocarcinoma and prostatic intraepithelial neoplasia. Histol Histopathol..

[26] Bostwick DG, Pacelli A, Blute M, Roche P, Murphy GP (1998). Prostate specific membrane antigen expression in prostatic intraepithelial neoplasia and denocarcinoma: a study of 184 cases. Cancer..

[27] Conteduca V, Aieta M, Amadori D, De Giorgi U (2014). Neuroendocrine differentiation in prostate cancer: current and emerging therapy strategies. Crit Rev Oncol Hematol..

[28] Beltran H (2014). Aggressive variants of castration-resistant prostate cancer. Clin Cancer Res..

[29] Conteduca V (2015). Impact of visceral metastases on outcome to abiraterone after docetaxel in castration-resistant prostate cancer patients. Future Oncol..

[30] De Giorgi U (2014). Early outcome prediction on 18F-fluorocholine PET/CT in metastatic castration-resistant prostate cancer patients treated with abiraterone. Oncotarget..

[31] De Giorgi U (2015). (18)F-fluorocholine PET/CT for early response assessment in patients with metastatic castration-resistant prostate cancer treated with enzalutamide. Eur J Nucl Med Mol Imaging..

[32] Perera M (2016). Sensitivity, specificity, and predictors of positive 68Ga-prostate-specific membrane antigenpositron emission tomography in advanced prostate cancer: a systematic review and meta-analysis. Eur Urol..

[33] Hirsch FR (2003). Epidermal growth factor receptor in non-small-cell lung carcinomas: Correlation between gene copy number and protein expression and impact on prognosis. J Clin Oncol..

[34] John T, Liu G, Tsao MS (2009). Overview of molecular testing in non-small-cell lung cancer: Mutational analysis, gene copy number, protein expression and other biomarkers of EGFR for the prediction of response to tyrosine kinase inhibitors. Oncogene..

